# Development of Cell Culture-Derived Hemagglutination Antigens for Japanese Encephalitis Virus Genotypes 1 and 3

**DOI:** 10.3390/v18080850

**Published:** 2026-08-03

**Authors:** Seong-In Lim, Dong-Kun Yang, Gyeong Hui Kwon, Hyobi Kim, Yoon-Gi Baek, Eun-Jung Kim, Eun Mee Park, Soo Hyun Moon, Min Ji Kim, Jae-Myung Kim

**Affiliations:** Viral Disease Division, Animal and Plant Quarantine Agency, Gimcheon 39660, Republic of Korea; yangdk@korea.kr (D.-K.Y.); kkhee234@korea.kr (G.H.K.); khb2015@korea.kr (H.K.); bygttaggg@korea.kr (Y.-G.B.); kejpo30@korea.kr (E.-J.K.); ebag0027@gmail.com (E.M.P.); msh103101@korea.kr (S.H.M.); mingming0525@korea.kr (M.J.K.); kimjm88@korea.kr (J.-M.K.)

**Keywords:** Japanese encephalitis virus, hemagglutination inhibition test, cell culture-derived antigen, genotype-specific surveillance

## Abstract

Conventional Japanese encephalitis virus (JEV) hemagglutination inhibition (HI) antigens are contingent on sucking mouse brain (SMB), and present ethical and standardization challenges. In this study, we established an alternative cell culture platform using LFBK cells to produce JEV genotype I (GI) and III (GIII) antigens. Viral replication and hemagglutination (HA) activity were evaluated in six cell lines. Although raw supernatants exhibited limited HA activity, 100-fold polyethylene glycol (PEG) 8000 concentration and binary ethylenimine (BEI) inactivation of LFBK cultures successfully resulted in high-titer antigens for the K95 (GI, 256 HAU), K87 (GIII, 64 HAU), and Nakayama (GIII, 1024 HAU) strains. Diagnostic validation with swine sera showed 100% concordance between the cell-derived Nakayama antigen and commercial SMB standard. Notably, cell-concentrated GI antigens (K95 and K87) demonstrated significantly higher sensitivity (*p* < 0.05) for several positive samples. These results indicate that the LFBK cell platform can effectively replace traditional SMB methods and provide an ethically sustainable and highly sensitive tool for genotype-specific JEV serosurveillance.

## 1. Introduction

Japanese encephalitis (JE) was first clinically documented in Japan in 1871 [1], and the causative agent, Japanese encephalitis virus (JEV), was subsequently isolated from a human patient in 1935 [2]. JEV, a member of the *Flaviviridae* family and *Orthoflavivirus* genus, is a leading cause of viral encephalitis [3].

JE is prevalent in extensive regions of the Pacific Ocean and Asia, with a high incidence and risk, seriously threatening the health of humans and animals. Countries with confirmed JE cases include India, Nepal, Pakistan, Sri Lanka, Myanmar, Laos, Vietnam, Malaysia, the Philippines, Singapore, China, Indonesia, Maritime Siberia, Japan, and Republic of Korea. Sporadic outbreaks have also been observed in the Western Pacific and northern Australia. JE is highly detrimental to human health and accounts for 70,000 human cases and 15,000 deaths annually [4,5]. The virus is transmitted by *Culex* mosquitoes between Ardeid waterbirds, such as the black-crowned night heron, and pigs as amplifying hosts; humans and horses are incidental dead-end hosts [6]. Vaccination of pigs and horses has been extensively implemented to reduce disease burden and viral circulation.

JEV has only one serotype, but can be classified into five genotypes according to the full-length genome sequence of the E gene, namely, genotypes I, II, III, IV, and V (GI, GII, GIII, GIV, and GV, respectively) [7]. Historically, GIII was the predominant lineage and served as the foundation for most currently available vaccines. However, GI has replaced GIII as the dominant circulating genotype in many Asian countries, and GV has recently emerged in several regions such as China and Republic of Korea. GIV caused the first large outbreak outside Southeast Asia in northern and eastern Australia in 2021–2022 [4,8]. Accumulating evidence indicates that antigenic differences among JEV genotypes, specifically regarding amino acid variations in the envelope (E) protein, significantly influence cross-neutralization efficacy and the protective breadth of vaccine-induced immunity. These antigenic disparities have verified cross-neutralization assays, which demonstrate reduced neutralizing antibody titers against heterologous genotypes compared to homologous strains [8,9,10,11].

Serological assays, such as the plaque-reduction neutralization test (PRNT), hemagglutination inhibition (HI) test, and enzyme-linked immunosorbent assay (ELISA), are critical in the diagnosis and surveillance of JEV infections [12]. The PRNT is considered the reference standard for measuring virus-specific neutralizing antibodies; however, it is labor-intensive and not readily applicable for large-scale surveillance. However, the HI test is widely used because of its simplicity, cost-effectiveness, and ability to rapidly process a substantial number of samples [13,14].

Conventional HI antigens for JEV have been prepared by sucking mouse brain (SMB), because JEV replicates at notably high concentrations within the mouse brain [15]. Although SMB-derived antigens have been traditionally used for JEV HI assays, research into cell-culture-based and recombinant protein platforms for JEV antigen production has been ongoing since the 1990s [16,17,18,19]. These alternative platforms were developed to address the limitations of SMB methods, including ethical concerns and variability in antigen quality.

In this study, we aimed to establish a cell culture-based system for producing hemagglutination antigens of JEV genotypes I and III, and to evaluate their applicability as genotype-specific HI antigens. By replacing conventional mouse brain-derived antigens with cell culture-derived preparations, this approach attempts to improve antigen standardization and provide a platform for comparative serological analysis across different JEV genotypes.

## 2. Materials and Methods

### 2.1. Virus Strains

Four JEV strains, representing different genotypes and origins, were used in this study. The K95 strain (NCCP No. 41320) is a GI virus isolated from *Culex tritaeniorhynchus* in Republic of Korea in 1995. The KV1899 strain (GenBank AY316157), also classified as GI, was isolated from the serum of a fattening pig in Gyeonggi Province, Republic of Korea, in 1999. To represent GIII, two strains were included: the K87 strain (GenBank AY585242) isolated from *Culex tritaeniorhynchus* in Wando, Jeonnam Province, Republic of Korea, in 1987, and the Nakayama strain (GenBank EF571853), a prototype GIII virus originally isolated from the cerebrospinal fluid (CSF) of a human patient in Japan in 1935 (Table 1).

### 2.2. Cell Culture

Vero cells (African green monkey kidney cell line, ATCC: CCL-81), BHK-21 cells (Baby Hamster Kidney-21, ATCC: C-13), LFBK cells (Fetal Porcine Kidney cell, CVCL-RX27), and NG108-15 cells (mouse neuroblastoma × rat glioma hybrid cell line, ATCC: HB-12317) were cultured in Dulbecco’s modified Eagle’s medium supplemented with 10% fetal bovine serum and 1% antibiotics. The cells were maintained at 37 °C in a humidified atmosphere containing 5% CO_2_. C6/36 cells (*Aedes albopictus* larval cell line, ATCC: CRL-1660) were cultured under identical conditions, except at an incubation temperature of 28 °C. *Sf*-9 cells (*Spodoptera frugiperda* ovarian tissue; ATCC: CRL-1711) were cultured in Grace’s medium (Gibco BRL, Grand Island, NY, USA) at 28 °C in a non-CO_2_ environment.

The Vero (ATCC: CCL-81), BHK21 (ATCC: CCL-10), NG108-15 (ATCC: HB-12317), and sf-9 (ATCC: CRL-1711) cell lines were purchased from the American Type Culture Collection (ATCC, Manassas, VA, USA). The LFBK cell line (CVCL-RX27) was kindly provided by the Foot-and-Mouth Disease (FMD) Vaccine Center at the Animal and Plant Quarantine Agency (APQA, Gimcheon, Republic of Korea).

### 2.3. Virus Propagation

To evaluate the susceptibility of various cell lines to JEV, six different cell lines were screened for viral propagation. Each cell line was seeded and cultured until 80–90% confluence was reached. The cells were then infected with JEV strains at a multiplicity of infection (MOI) of 0.1. Following viral inoculation, the cells were incubated for 1 h at their respective optimal temperatures—37 °C for mammalian cell lines and 27 °C for *Sf9* insect cells—to allow for viral adsorption. Subsequently, the inoculum was replaced with a maintenance medium containing 2% fetal bovine serum, and the cultures were maintained at 37 °C. The infected cell cultures were monitored daily for the development of cytopathic effects (CPE). CPE induced by JEV was typically observed starting from 3 days post-infection (DPI), and the culture supernatants were harvested at 5 DPI, at which point approximately 90% CPE was attained.

### 2.4. Virus Titration Assay

Viral infectivity titers were determined using a 50% tissue culture infectious dose (TCID_50_) assay on susceptible cell monolayers. Briefly, cells were seeded in 96-well microtiter plates at a density of 1.5 × 10^4^ cells per well and incubated at 37 °C in a 5% CO_2_ atmosphere until they reached approximately 80–90% confluence. The virus stocks were ten-fold serially diluted (10^−1^ to 10^−8^) in maintenance medium supplemented with 2% fetal bovine serum (FBS). Following the removal of the growth medium, 100 µL of each viral dilution was inoculated into eight replicate wells. The plates were incubated for 5 to 7 days to allow the development of virus-specific cytopathic effects (CPE). Notably, the incubation temperature was adjusted according to the cell line used: mammalian cell lines, including Vero, BHK-21, LFBK, and NG108, were maintained at 37 °C in a 5% CO_2_ atmosphere, whereas the mosquito-derived C6/36 cell line was incubated at 28 °C without CO_2_. The final TCID_50_ titers were calculated using the Reed–Muench method and expressed as log_10_TCID_50_/mL. Wells inoculated with maintenance medium served as negative controls to ensure the validity of the CPE observations. To ensure the reproducibility of the results, all experiments were performed in triplicate. The viral titration for each cell line was conducted using three independent biological replicates.

### 2.5. Production and Concentration of JEV HI Antigens

K95, K87, and Nakayama strains used for HI testing were propagated in LFBK cells under standard culture conditions. When approximately 90% cytopathic effects were observed, the infected cell cultures in 175 cm^2^ flasks were subjected to three freeze–thaw cycles. The harvested supernatant was inactivated at 37 °C for 10 h using 2 Mm binary ethylenimine (BEI), following established protocols [19,20,21].

To confirm complete viral inactivation, the treated supernatant was dialyzed and subsequently inoculated into LFBK cells; no viral replication was detected.

After checking the inactivation of viruses, the inactivated strains were concentrated 100-fold with polyethylene glycol 8000 (PEG 8000; Sigma-Aldrich, Mumbai, MA, USA) for further evaluation. HA titers of the unconcentrated and concentrated antigens were measured [21]. Sodium chloride was added to obtain a final concentration of 0.5 M and PEG 8000 was added at 4 °C for 10 h for the precipitation of virus and then centrifuged at 10,000× *g* for 30 min. The results pellet was carefully resuspended in a minimal volume of PBS (pH 7.4), representing a 100-fold concentration of the original supernatant. The concentrated antigen was aliquoted and stored at −80 °C until further use.

### 2.6. Hemagglutination (HA) Assay

The hemagglutinating activity and titers of the JEV antigens were determined by HA assays, following the standardized protocol originally described by Clarke and Casals (1958), with minor modifications [15]. Briefly, the JEV antigens prepared from each strain were serially diluted two-fold in a virus diluent (pH 9.0, borate-buffered saline supplemented with 0.4% bovine serum albumin) in 96-well V-bottom microtiter plates. Fifty microliters of 0.33% suspension of washed goose red blood cells (RBCs) in dextrose–gelatin-veronal (DGV) buffer were added to each well containing the diluted antigen. For the HA assay, titers were measured in triplicate for both the raw culture supernatant and the concentrated samples.

### 2.7. Hemagglutination Inhibition (HI) Test

Pig sera were inoculated with JEV GI and GV antigens. The HI test was performed using the HI test with K95, KV1899, K87, and Nakayama strains. An HI test was performed in 96-well microplates, using slightly modified standard methods [15,19]. Using the sucrose/acetone extraction method, JEV GI antigens were prepared from the brains of suckling mice infected with the KV1899 strain. JEV GI antigens were prepared by concentrating the virus obtained from LFBK cells infected with K95. Two JEV GIII antigens were also prepared using the same method in LFBK cells infected with the Nakayama and K87 strains.

As an HI test procedure, 100 µL of the serum was mixed with 900 µL of 14.5% kaolin (Sigma, West Hollywood, CA, USA) to remove non-specific inhibitors and incubated for 30 min. After pipetting, kaolin was removed by centrifugation at 3600 rpm for 15 min. The resultant clear supernatant was mixed with 20 µL of packed goose erythrocytes to remove any natural agglutinins. After incubation at 37 °C for 1 h, the treated sera were separated from the goose erythrocytes by centrifugation. The treated sera (25 µL) were diluted 2-fold from 1:20 to 1:10,240 in round-bottom 96-well microplates and reacted with eight HA units of the JEV antigen. After incubation at 37 °C for 1 h, 50 µL of 0.33% goose erythrocytes was added, and the microplates were incubated for 30 min at 37 °C. To confirm the test’s reliability, positive and negative JEV-infection pig control sera were used in the HI test. The HI titer was expressed as the reciprocal of the highest dilution of serum that showed complete HI. The HI test using pig sera was performed in triplicate for each serum aliquot to ensure measurement accuracy.

### 2.8. Preparation of Pig Sera

Eight-week-old JEV-negative piglets were used to prepare JEV-positive and JEV-negative control sera. To produce genotype-specific antisera against GI and GV, 12 piglets were randomly assigned to two groups (*n* = 6 per group). The piglets were allowed a one-week acclimatization period prior to the experiments. The piglets were housed in a biosafety level 2 (BSL-2) animal facility with controlled temperature and humidity. They were provided with ad libitum access to feed and water throughout the experimental period. To ensure objectivity, the investigators were blinded to the group allocation during the experiments and the subsequent serological analysis.

Each group was immunized twice at two-week intervals with inactivated GI or GV JEV antigens. Two piglets were intramuscularly injected with PBS as a negative control, whereas G1- and G5-immunized piglets received the respective antigens via the intramuscular route. Animals were immunized twice at two-week intervals. Three weeks after the second immunization, blood samples were collected, and the final sera were prepared and used for subsequent serological assays.

In this study, ‘positive’ serum samples were defined as those exhibiting anti-JEV antibody titers equal to or greater than the cut-off value determined by the commercial HI diagnostic kit (≥1:10), indicating prior exposure or vaccination. Conversely, ‘negative’ samples were defined as those with no detectable anti-JEV antibody activity (<1:10), confirming the absence of JEV-specific antibodies.

All animal experiments were approved by the Animal and Plant Quarantine Agency of Korea (approval code: APQA 2024-756). The piglets were monitored daily for signs of pain or illness. Humane endpoints were established as follows: a weight loss of >20%, severe lethargy, or inability to consume food/water. In such cases, piglets were humanely euthanized. No unexpected adverse events occurred during the study.

### 2.9. Statistical Analysis

Statistical analyses were performed using Microsoft Excel ver.10 (Microsoft Corp., Redmond, WA, USA). The mouse brain-derived Nakayama strain, used as the standard antigen in a commercially available JEV HI diagnostic kit (Median Diagnostics, Chuncheon, Republic of Korea), was designated as the reference standard for all comparative evaluations. The statistical analysis was conducted in two stages. To verify the consistency of the production platform, a paired Student’s *t*-test was used to compare HI titers obtained using the commercial standard and the newly developed cell culture-based Nakayama antigen. Second, the diagnostic efficacy of the concentrated GI strains (K87 and K95) was evaluated against the commercial Nakayama standard. All HI titers were log2 transformed before analysis to ensure data normality. Data are presented as mean ± standard deviation (SD). A *p*-value of less than 0.05 was considered statistically significant.

## 3. Results

### 3.1. Differential Replication Efficiency of JEV Strains Across Various Cell Lines

To evaluate host cell susceptibility and replication kinetics of JEV, two G1 strains (KV1899 and K95) and two GIII strains (K87 and Nakayama) were inoculated into six different cell lines. As presented in Table 2, the viral titers, expressed as log_10_TCID_50_/mL, varied significantly depending on both the viral strain and host cell type. Among the GI strains, KV1899 exhibited the lowest replication levels in NG108 and Sf9 cells (2.7 log_10_TCID_50_/mL), and reached its peak titer in C6/36 cells (5.5 log_10_TCID_50_/mL). In contrast, the K95 strain showed the least efficiency in Vero cells (4.0 log_10_TCID_50_/mL) and demonstrated robust growth in BHK-21 (7.4 log_10_TCID_50_/mL) and LFBK cells (7.0 log_10_TCID_50_/mL). Regarding the GIII strains, K87 showed minimal growth in Sf9 cells (3.0 log_10_TCID_50_/mL), and reached substantially higher titers in BHK-21 (7.3 log_10_TCID_50_/mL) and LFBK cells (7.5 log_10_TCID_50_/mL). Similarly, the Nakayama strain exhibited its lowest titers in Vero and Sf9 cells (3.0 log_10_TCID_50_/mL), whereas replication was optimal in BHK-21 (7.8 log_10_TCID_50_/mL), followed by NG108 (6.7 log_10_TCID_50_/mL) and LFBK cells (6.5 log_10_TCID_50_/mL). Overall, these results indicate that BHK-21 and LFBK cells generally facilitated high-titer replication of most JEV strains, whereas Sf9 cells consistently yielded the lowest viral productivity across all tested genotypes.

### 3.2. Evaluation of Hemagglutinating (HA) Activity from Cell Culture Supernatants

**Figure 1:**
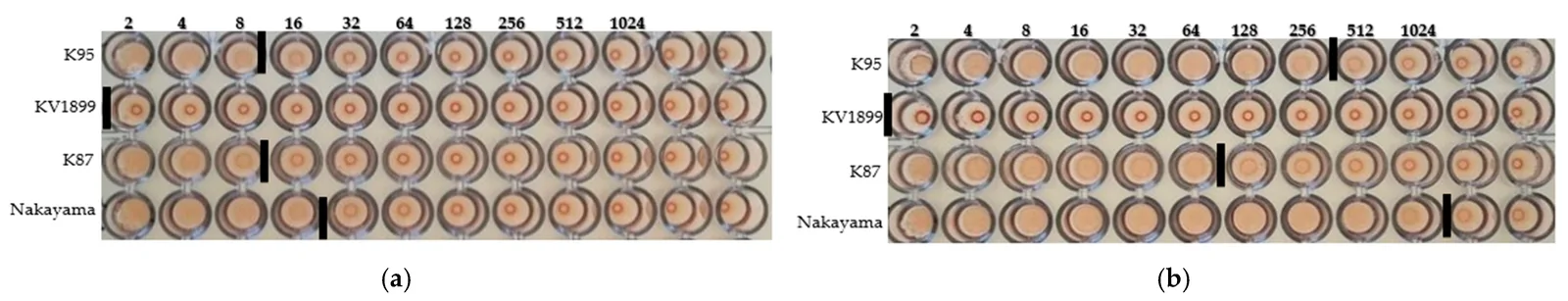
Hemagglutination (HA) titers of raw (**a**) and 100-fold concentrated supernatants (**b**) from JEV-infected LFBK cells. The formation of a diffuse reddish coat indicates positive HA activity, while a compact pellet (button) at the center indicates negative activity. Note the absence of HA activity in KV1899 even after 100-fold concentration, in contrast to the significant titer increase in K95, K87, and Nakayama strains. Black vertical bars indicate the endpoint used for determination of the HA titer.

**Figure 2:**
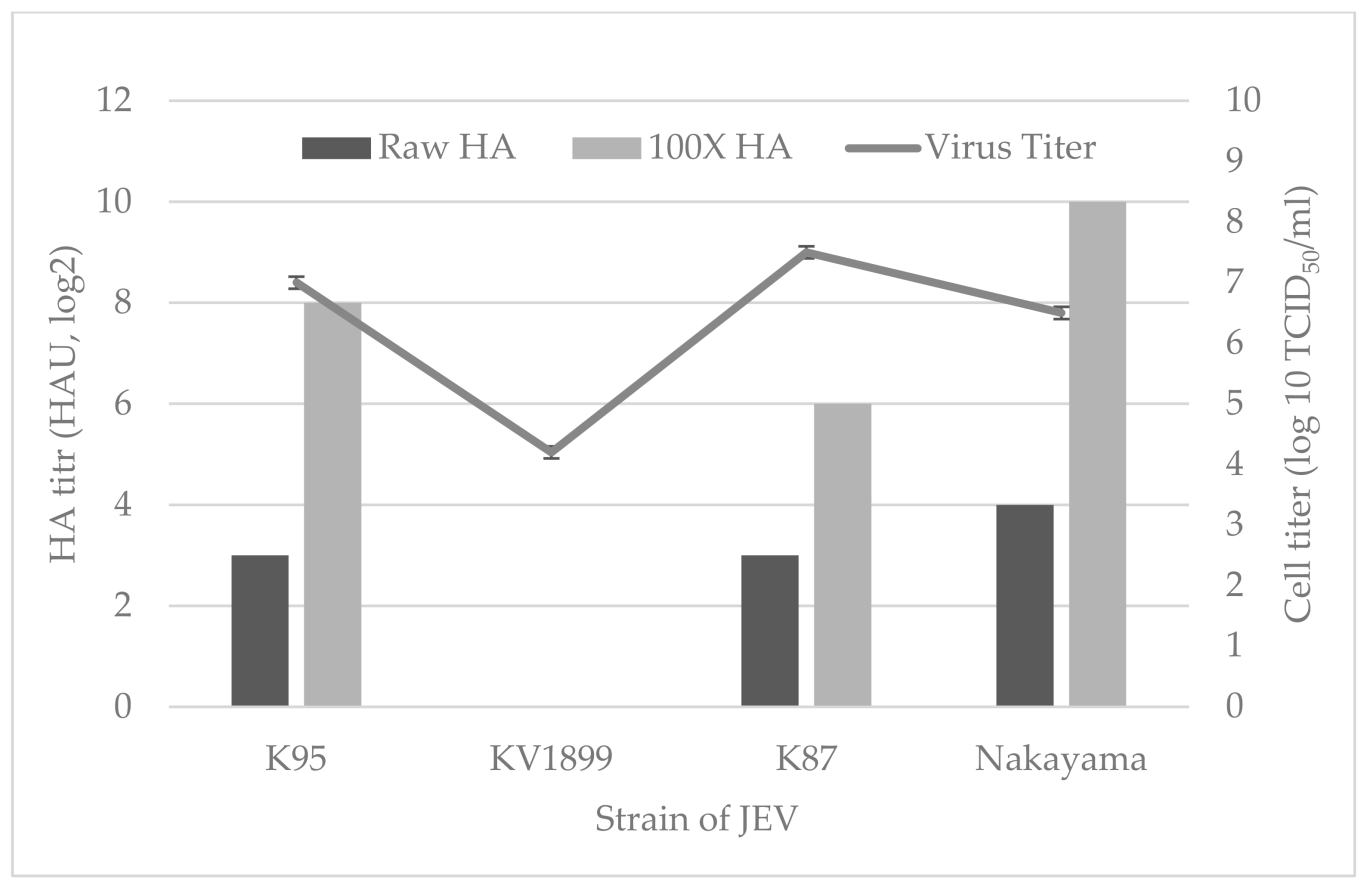
Viral replication efficiency and HA titers of JEV strains before and after PEG concentration in LFBK cells. Bars represent the HA titers of raw supernatants (dark gray) and 100-fold concentrated antigens (light gray) measured on the left y-axis. The line graph indicates the viral infectivity titers (log 10 TCID_50_/mL) measured on the right y-axis. Data represent the mean ± standard deviation (SD) from three independent experiments (*n* = 3) for virus titers, with error bars included. HA titers were also measured in triplicate (*n* = 3), and all replicate measurements yielded identical values. All data were obtained from JEV-infected LFBK cell cultures.

The hemagglutination activity of the JEV strains was evaluated using both raw culture supernatants and 100-fold concentrated preparations. In the unconcentrated supernatants, the GI strain K95 exhibited HA titers ranging from 8 to 16 HA units (HAU) in Vero, BHK-21, and LFBK cells, whereas no HA activity was observed in the KV1899 strain across all six cell lines. Among GIII strains, K87 showed the lowest HA activity in BHK-21 (2 HAU) and LFBK (8 HAU) cells. In contrast, the Nakayama strain demonstrated activity in BHK-21 (32 HAU) and LFBK (16 HAU) cells but did not show HA activity in the remaining cell lines. To further enhance antigen concentration, the supernatants were concentrated 100-fold using PEG 8000. At increased concentrations, KV1899 did not exhibit detectable HA activity in all cell lines. However, significantly elevated HA titers were observed in LBFK cells compared to those of other strains. K95 was 256 HAU, K87 was 64 HAU, and the Nakayama strain showed the highest activity at 1024 HAU (Table 3 and Figure 1 and Figure 2).

### 3.3. Comparative Analysis of JEV Antigens Obtained from Mouse Brain and Cell Culture Platforms

**Figure 3:**
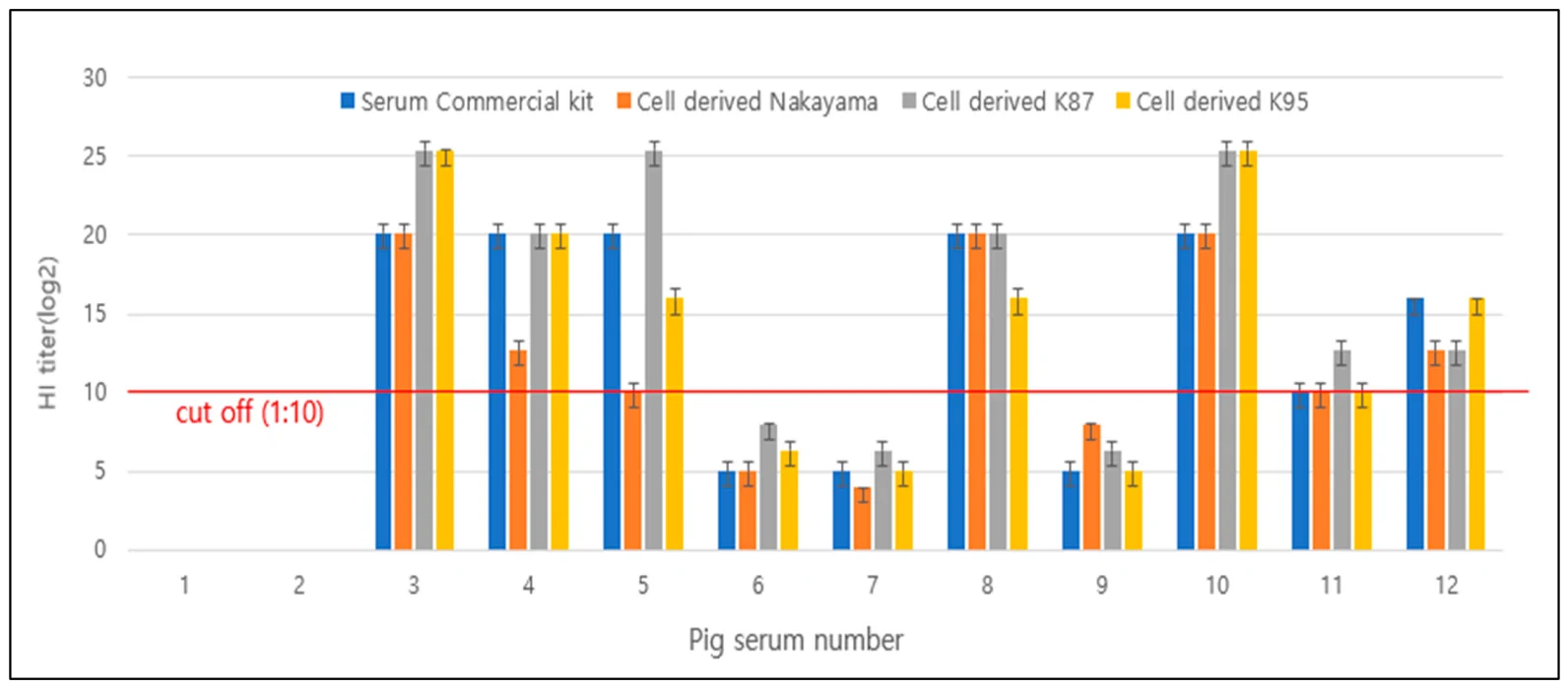
Comparison of HI antibody titers in swine sera using a commercial mouse brain-derived antigen and cell-concentrated JEV antigens. The HI antibody titers of 12 swine serum samples were evaluated to validate the diagnostic performance of newly developed cell-concentrated antigens (Nakayama, K87, and K95) against the established commercial standard antigen (Nakayama strain obtained from mouse brain; Median Diagnostic, South Korea). Lanes 1 and 2 serve as negative controls, whereas lanes 3 to 12 represent sera from inoculated animals. Among the inoculated individuals, subjects 6, 7, and 9 showed HI antibody titers of less than 10-fold and were ultimately judged as negative. All three cell-derived antigens showed diagnostic concordance compared with the commercial kit, and all demonstrated statistical significance regarding HI antibody titers (*p* < 0.05). The 12 swine serum samples were tested in triplicate (*n* = 3), and the graph displays the mean antibody titers with error bars representing the standard deviation (SD).

To validate the diagnostic reliability of the cell-derived antigens, we initially conducted a comparative analysis using the Nakayama strain, specifically comparing the commercial standard antigen (Nakayama-SMB from Median Diagnostics) with our cell culture-derived version (Nakayama-Cell). Using 12 swine serum samples, classified as JEV antibody-negative (*n* = 5, <1:10) and JEV antibody-positive (*n* = 7, ≥1:10) samples, both antigens showed 100% diagnostic concordance (Figure 3). Statistical analysis confirmed that there was no significant difference in HI titers between the commercial standard and cell-derived Nakayama antigen. Subsequently, the performance of the cell-concentrated GI strains K87 and K95 was evaluated against the same commercial standard (Nakayama-SMB). Although all samples remained within a one-fold dilution range (1 log2 unit), providing functional equivalence, GI antigens exhibited a statistically significant increase in sensitivity (*p* < 0.05) for several JEV antibody-positive samples. This indicates that the cell culture-derived antigens meet the rigorous diagnostic criteria of existing commercial kits, as well as provide potentially enhanced detection capabilities for circulating JEV genotypes.

## 4. Discussion

In Republic of Korea, thousands of cases and hundreds of deaths occurred annually prior to the introduction of the JE vaccine in the early 1970s. Following the expansion of the national immunization program in the 1980s, there has been a notable decrease in its incidence. However, since 2010, case reports have persisted, ranging from 10 to 40 cases per year. Between 2010 and 2023, 275 cases and 40 deaths have been recorded [22,23].

The JEV strains are classified into five genotypes based on their nucleotide sequence homology [7]. GI viruses were introduced into Republic of Korea in 1993 and were subsequently replaced by GIII viruses with the dominant circulating JEV genotype. The JE GV virus was first reported in a *Culex* mosquito in 2011 [24].

Pigs raised in farms have been vaccinated against JE since 1990. Since 2001, serosurveillance for JEV has been conducted in pig populations to evaluate herd immunity in sows and monitor JEV in fattening pigs [21,25].

Since 2007, no official notifications of an outbreak in the Republic of Korean pig population have been reported since 2007 [25]. However, the incidence of JE in humans is increasing annually [10,22].

Severe climate change and global warming may notably affect the incidence of vector-borne diseases, including JEV infections, in humans and animals. Therefore, veterinary authorities require numerous diagnostic tools to survey vector-borne diseases [21]. The lack of overt symptoms in most veterinary hosts contributes to the silent spread of JEV in the endemic areas. Therefore, surveillance systems that rely solely on clinical presentation are inadequate [11]. Monitoring JEV activity in mosquito vectors and domestic pigs enables health authorities to accurately predict risks and implement timely vector control measures when risks are detected. In practice, certain countries monitor seroconversion in sentinel animals, such as pigs, dogs, and chickens [26,27,28,29,30,31].

The detection of antibodies against JEV is crucial for both epidemiological surveillance and the evaluation of vaccine efficacy. Among the various serological methods available, the PRNT is regarded as the reference standard because of its high specificity and sensitivity in measuring neutralizing antibodies. However, the PRNT method is inherently time-consuming, technically demanding, and requires live viruses, limiting its practical application in routine diagnostics and large-scale studies. In contrast, the HI assay provides a technically simple, rapid, and cost-effective approach. The HI assay can serve as a practical tool for large-scale serological surveys and vaccine efficacy studies, providing a balance between accuracy and feasibility [12,13,14].

JEV is cultivated and propagated in cell culture systems, and the JEV supernatant does not exhibit hemagglutinating activity. Therefore, hemagglutinating antigens prepared from sucking mouse brain (SMB) infected with JEV using the sucrose–acetone extraction method have been used to examine the immune status of JEV in several animals [21]. For the HI test, hundreds of sucking mice are injected with the virus and killed to obtain the antigen, which remains a controversial practice in relation to laboratory animal welfare [26]. The sucrose–acetone extraction method is biologically hazardous to manufacturers [21]. Generally, the virus produced in newborn mice results in a high titer; however, the mouse brain material must be removed with several treatments, such as acetone.

For JEV GV serological monitoring, Yang et al. developed a cell-derived HI antigen that enhances the diagnostic accuracy and provides a replacement for traditional murine-derived antigen production [19].

In the present study, we evaluated the replication efficiency of JEV across six different cell lines to develop alternative cell-derived HI antigens for GI and GIII, effectively mitigating the ethical and biosafety concerns associated with traditional SMB-derived antigens. Among the tested cell lines, the LFBK cell line exhibited the highest viral productivity for several JEV strains. Although raw cell culture supernatants exhibited limited or variable HA activity, subsequent PEG 8000 concentration and BEI inactivation effectively resulted in high-titer functional HI antigens, demonstrating the strong potential of cell culture-based platforms for diagnostic applications.

Among the six cell lines screened, LFBK cells were selected as the optimal platform for antigen production. While Vero and BHK-21 cells supported robust viral replication, LFBK cultures consistently yielded the highest HA titers, particularly following concentration. We hypothesize that LFBK cells, being of porcine origin, a natural amplifying host for JEV, provide a superior microenvironment for viral protein processing. Specifically, LFBK cells may facilitate the correct folding and post-translational glycosylation of the E protein, which is critical for maintaining the structural integrity and HA activity of JEV particles. Unlike other cell lines where high-titer replication did not necessarily correlate with HA activity, the LFBK platform appears uniquely capable of preserving the hemagglutinating functionality of JEV antigens during the concentration process.

A notable finding of our study was that robust viral replication did not consistently correlate with HA activity. For example, despite active replication, certain conditions did not produce detectable HA titers. According to previous studies, this discrepancy can be attributed to several critical factors, including structural alterations in the envelope (E) protein, host-specific differences in glycosylation patterns, discrepancies between intracellular and extracellular viral particles, and the strict pH dependence required for flavivirus HA stability. Notably, our findings regarding the KV1899 strain present a notable contradiction to the previous literature. Yang et al. reported that the KV1899 strain propagated in Vero cells exhibited higher HA titers than those obtained through traditional SMB inoculation. In contrast, our study demonstrated that KV1899 replicated more efficiently in other cell lines than in Vero cells, and notably did not exhibit any detectable HA activity in both raw supernatants and 100-fold concentrated preparations. Although the exact viral titer achieved in Vero cells was not explicitly detailed in a previous study, our KV1899 preparations resulted in relatively low titers in Vero cells. There are several plausible explanations for this discrepancy. First, a viral concentration threshold is required to initiate visible hemagglutination. Second, although designated as the same KV1899 strain, genetic drift or laboratory-specific passages may have introduced critical mutations within the E protein, thereby altering its hemagglutination properties.

To validate the clinical utility of our alternative platform, we evaluated the diagnostic reactivity of the newly developed cell-derived antigen against a panel of JEV-positive swine sera, using a widely accepted commercial SMB-derived antigen (Median Diagnostics, South Korea) as the reference standard. The cell-derived antigens demonstrated excellent diagnostic concordance and reactivity profiles, comparable to those of the commercial reference, statistically confirming their viability as alternative diagnostic reagents.

Although the sample size (*n* = 12) was sufficient to demonstrate the diagnostic concordance between the cell-derived and SMB-derived antigens in this proof-of-concept study, further large-scale validation across diverse field-collected swine populations is essential. To ensure statistical rigor, we confirmed the normality of the HI titer distribution, justifying the use of the paired Student’s *t*-test for evaluating the performance of the newly developed cell-based antigens against the commercial reference. Future studies will focus on expanding the sample size to enhance the statistical power and assess batch-to-batch consistency.

Several technical challenges must be addressed to facilitate the commercialization and mass production of cell-derived HI antigens. First, the optimization of upstream bioprocesses is crucial for preventing undesirable mutations in the E protein during large-scale manufacturing. Second, rigorous standardization and batch-to-batch reproducibility protocols must be established. SMB-derived antigens provide high antigenic stability with minimal variation; however, their cell-derived counterparts are inherently susceptible to altered glycosylation patterns depending on the host cell line, as well as conformational changes in the envelope protein during sequential passage. Therefore, further systematic studies focusing on the structural stability and glycan profiling of cell-propagated JEV antigens are necessary to ensure reliable and standardized diagnostic performance in routine veterinary surveillance.

While this study successfully validated the LFBK-based antigen production platform, future research is required to ensure consistent batch-to-batch reproducibility. For mass production, we intend to implement standardized upstream bioprocesses and structural stability assessments, such as glycan profiling and conformational analysis of the E protein, to ensure that cell-derived antigens maintain high-titer hemagglutination activity across independent production cycles.

## 5. Conclusions

In conclusion, this study successfully established a sustainable and standardized cell culture-based platform using LFBK cells to produce high-titer JEV hemagglutination antigens for genotypes I and III, thereby providing an effective alternative to the traditional sucking mouse brain (SMB)-derived antigens. Using the PEG 8000 concentration and BEI inactivation, we developed functional cell-derived antigens that completely mitigated the ethical concerns and biological safety hazards associated with murine models.

Diagnostic validation using swine sera confirmed that the cell-derived antigens achieved 100% diagnostic concordance and reactivity profiles comparable to those of existing commercial gold standards. Additionally, the cell-concentrated GI antigens demonstrated notably enhanced sensitivity in detecting antibodies in positive samples, underscoring their potential for improved monitoring of currently circulating JEV genotypes. Although further large-scale validation and optimization are required to address batch-to-batch reproducibility and structural stability during upstream bioprocessing, this cell-derived antigen platform represents a crucial advancement toward establishing a robust, ethically compliant veterinary surveillance infrastructure for vector-borne diseases.

## Figures and Tables

**Table 1:** Summary of Japanese encephalitis virus (JEV) strains used for antigen preparation and serological assays.

Strain	Genotype	Origin/Source	Location
K95	GI	*Culex tritaeniorhynchus*	South Korea
KV1899	GI	Swine (Fattening pig)	Gyeonggi, SK
K87	GIII	*Culex tritaeniorhynchus*	Wando, SK
Nakayama	GIII	Human (CSF)	Japan

**Table 2:** Titers of JEV strains according to various cell lines.

Virus		Cell Line (log_10_TCID_50_/mL) *					
Genotype	Strain	Vero	BHK-21	LFBK	NG108	C6/36	Sf9
GI	K95	4.0 ± 0.1	7.4 ± 0.2	7.0 ± 0.1	6.7 ± 0.2	6.5 ± 0.1	4.3 ± 0.1
	KV1899	3.5 ± 0.1	4.0 ± 0.2	4.2 ± 0.1	2.7 ± 0.1	5.5 ± 0.1	2.7 ± 0.2
GIII	K87	5.5 ± 0.1	7.3 ± 0.0	7.5 ± 0.1	5.2 ± 0.0	5.3 ± 0.1	3.0 ± 0.1
	Nakayama	3.0 ± 0.1	7.8 ± 0.1	6.5 ± 0.1	6.7 ± 0.1	6.3 ± 0.1	3.0 ± 0.2

* Values represent the mean ± standard deviation (SD) from three independent experiments (*n* = 3).

**Table 3:** Hemagglutination (HA) titers of JEV strains according to cell lines and PEG concentration.

Virus		Preparation	Cell Line (HAU) *					
Genotype	Strain		Vero	BHK-21	LFBK	NG108	C6/36	Sf9
GI	K95	Raw Supernatant	8	16	8	0	0	0
		100× Conc.	0	0	256	0	0	0
	KV1899	Raw Supernatant	0	0	0	0	0	0
		100× Conc.	0	0	0	0	0	0
GIII	K87	Raw Supernatant	0	2	8	0	0	0
		100× Conc.	0	0	64	0	0	0
	Nakayama	Raw Supernatant	0	32	16	0	0	0
		100× Conc.	0	0	1024	0	0	0

* Hemagglutination (HA) titers were measured in triplicate (*n* = 3), and all replicate measurements yielded identical values.

## Data Availability

The data presented in this study are available upon request from the corresponding authors. These data are not publicly available because of patent applications in Republic of Korea.

## References

[1] Kuni G. (2022). Contrasting the Practices of Virus Isolation and Characterization between the Early Period in History and Modern Times: The Case of Japanese Encephalitis Virus. Viruses.

[2] Hayashi V.M. (1934). Ubertragung des virus von encephalitis epidemica afu affen. Proc. Acad. Tokyo.

[3] Vanghn D.W., Hoke C.H. (1992). The epidemiology of Japanese encephalitis: Prospects for prevention. Epidemiol. Rev..

[4] Campbell G.L., Hills S.L., Fischer M., Jacobson J.A., Hoke C.H., Hombach J.M., Marfin A.A., Solomon T., Tsai T.F., Tsu V.D. (2011). Estimated global incidence of Japanese encephalitis: A systematic review. Bull. World Health Organ..

[5] Xia Q., Yang Y., Zhang Y., Zhou L., Ma X., Xiao C., Zhang J., Li Z., Liu K., Li B. (2023). Shift in dominant genotypes of Japanese encephalitis virus and its impact on current vaccination strategies. Front. Microbiol..

[6] Mackenzie J.S., Williams D.T., van den Hurk A.F., Smith D.W., Currie B.J. (2022). Japanese Encephlitis Virue: The Emergence of Genotype IV in Australia and Its Potential Endemicity. Viruses.

[7] Chen W.-R., Tesh R.B., Rico-Hesse R. (1990). Genetic Variation of Japanese Encephalitis Virus in Nature. J. Gen. Virol..

[8] Li Q., Mishra H., Kain K.C., Wang R. (2025). The hidden threats posed by Japanese encephalitis virus genotype V. J. Virol..

[9] Kang B.-K., Hwang J.-M., Moon H., Han S.-K., Kim J.-M., Yang D.-K., Park B.-K., Song D. (2026). Comparison of the antigenic relation between Japanese encephalitis virus genotype. Clin. Exp. Vaccine Res..

[10] Lee A.-R., Song J.M., Seo S.-U. (2022). Emerging Japanese Encephalitis Virus Genotype V in Republic of Korea. J. Microbiol. Biotechnol..

[11] Park J.-Y., Lee H.-M. (2025). Managing Japanese Encephalitis Virus as a Veterinary Infectious Disease Through Animal Surveillance and One Health Control Strategies. Life.

[12] WOAH (World Organisation for Animal Health) (2021). Manual of Diagnostic Tests and Vaccines for Terrestrial Animals.

[13] Li C., Wan J., Wang D., Xiao L., Li X., Zhang C., Wang Z. (2025). Comparative Analysis of Hemagglutination Inhibition and Plaque Reduction Neutralization Tests for Japanese Encephalitis Virus Antibody Detection. Viruses.

[14] Mansfield K.L., Hernandez-Triana L.M., Banyard A.C., Fooks A.R., Johnson N. (2017). Japanese encephalitis virus infection, diagnosis and control in domestic animal. Vet. Microbiol..

[15] Clarke D.H., Casals J. (1958). Techniques for hemagglutination and hemagglutination-inhibition with arthropod-borne viruses. Am. J. Trop. Med. Hyg..

[16] Hasegawa H., Satake Y., Kobayashi Y. (1990). Effect of Cytokines on Japanese Encephalitis Virus Production by Human Monocytes. Microbiol. Immunol..

[17] Konishi E., Fujii A., Mason P.W. (2001). Generation and Characterization of a Mammalian Cell Line Continuously Expressing Japanese Encephalitis Virus Subviral Particles. J. Virol..

[18] Yasuda A., Kimura-Kuroda J., Ogimoto M., Miyamoto M., Sata T., Sato T., Takamura C., Kurata T., Kojima A., Yasui K. (1990). Induction of Protective Immunity in Animals Vaccinated with Recombinant Vaccinia Viruses That Express PreM and E Glycoproteins of Japanese Encephalitis Virus. J. Vriol..

[19] Yang D.-K., Park G.-N., Lee J.-Y., Lee H.J., Kwon G.H., Cho Y.S. (2025). Development of a novel antigen for hemagglutination inhibition of Japanese encephalitis virus genotype 5. Korean J. Vet. Res..

[20] Larghi O.P., Nebel A.E. (1980). Rabies virus inactivation by binary ethylenimine: New method for inactivated vaccine production. J. Clin. Microbiol..

[21] Yang D.K., Kim H.-H., Nah J.-J., Lee K.-W., Song J.-Y. (2012). Binary Ethylenimine Inactivated Japanese Encephalitis Virus Antigen Reveals Hemagglutination. J. Vet. Med..

[22] Cho S.-R., Lee Y.-J., Han M.G., Kim H.M. (2025). Complete Genome Sequences of Human Japanese Encephalitis Virus Genotype V Isolates in Korea Reveal Genotype-Specific Amino Acid Signatures. Pathogens.

[23] Yun B.-R., Kwon J.-Y., Noh B.-E., Cho S., Kwak D., Lee H.I. (2025). Genetic shifts of Japanese encephalitis virus (JEV) in mosquitoes in the Republic Korea, 2017–2022. PLoS Negl. Trop. Dis..

[24] Li M.-H., Fu S.-H., Chen W.-X., Wang H.-Y., Guo Y.-H., Liu Q.-Y., Li Y.-X., Luo H.-M., Duo Ji D.Z., Ye X.-M. (2011). Genotype V Japanese Encephalitis Virus Is Emerging. PloS. Negl. Trop. Dis..

[25] Nah J.-J., Yang D.-K., Kim H.-H., Song J.-Y. (2015). The present and future of veterinary vaccines for Japanese encephalitis in Korea. Clin. Exp. Vaccine Res..

[26] Cha G.-W., Lee E.J., Lim E.-J., Sin K.S., Park W.W., Jeon D.Y., Han M.G., Lee W.-J., Choi W.-Y., Jeong Y.E. (2015). A Novel Immunochromatographic Test Applied to a Serological Survey of Japanese Encephalitis Virus on Pig Farms in Korea. PLoS ONE.

[27] Shimoda H., Ohno Y., Mochizuki M., Iwata H., Okuda M., Maeda K. (2010). Dogs as Sentinels for Human Infection with Japanese Encephalitis virus. Emerg. Infect. Dis..

[28] Nitatpattana N., Le Flohic G., Thongchai P., Nakgo K., Palaboodeewat S., Khin M., Barbazan P., Ykosan S., Gonzalez J.-P. (2011). Elevated Japanese Encephalitis Virus Activity Monitored by Domestic Sentinel Piglets in Thailand. Vector Borne Zoonotic Dis..

[29] Shimoda H., Inthong N., Noguchi K., Terada Y., Nagao Y., Shimojima M., Takasaki T., Rerkamnuaychoke W., Maeda K. (2013). Development and application of an indirect enzyme-linked immunosorbent assay for serological survey of Japanese encephalitis virus infection in dogs. J. Virol. Methods.

[30] Yap G., Lim X.F., Chan S., How C.B., Humaid M., Yeo G., Mailepessov D., Kong M., Lai Y.L., Okumura C. (2019). Serological evidence of continued Japanese encephalitis virus transmission in Singapore nearly three decades after end of pig farming. Parasites Vectors.

[31] Ahmed W., Gebrewold M., Williams D.T., Wang J., Smith W.J., Starick L.G., Fogarty R., Richards K., Simpson S.L. (2025). Surveillance of Japanese encephalitis virus in piggery effluent and environmental samples: A complementary tool for outbreak detection. Appl. Environ. Microbiol..

